# A Letter from Savannah

**Published:** 1885-07

**Authors:** R. J. Nunn

**Affiliations:** Savannah, Ga.


### Correspondence.

### A LETTER FROM SAVANNAH.

JOURNALIZING THE PROCEEDINGS OF THE ASSOCIATION—SHALL THE ASSOCIATION BE LOCATED—NECESSITY FOR FREE INTERCHANGE OF OPINIONS—THE ASSOCIATION IN THE PAST, ETC.

*Editors Atlanta Medical and Surgical Journal:*

The journalizing of the proceedings of the Medical Association of Georgia is the most important measure which has come before the Association for years. That now is fixed and determined, and with the consummation of the arrangements with the ATLANTA MEDICAL AND SURGICAL JOURNAL, the Association will enter upon a new era in its existence, equipped more in accordance with the requirements of the age.

### THE BUSINESS BEFORE THE COMMITTEE.

But now comes a point to be determined upon which hangs the very existence of the body as a State organization: "Shall the Medical Association of Georgia be located?" Than this no more momentous question has ever been put before the Association for solution, and, acting with a wise caution, the Association at its last meeting appointed a committee, composed of members from different sections of the State, to examine into the matter and report at the next meeting.

### NECESSITY FOR A FREE INTERCHANGE OF OPINIONS.

Having to report upon a subject of so much interest to the Association, it is proper that the committee should not only consult within its own membership, but that it should likewise draw counsel, advice and suggestions from the other members of the Association. These views should be calm and deliberate, and should not be influenced by the heat of debate, or any spirit of partisanship, but should look solely to the permanent benefit of the Association. To attain the free interchange of opinions just spoken of by means of private correspondence would be well-nigh an impossibility, and some other medium of intercommunication becomes necessary. Naturally THE JOURNAL, the organ of the Association, suggests itself as the most appropriate, and certainly there can be no objection to the use of its columns for this purpose.

### THE ASSOCIATION IN THE PAST.

The Association in the years gone by could scarcely be called peripatetic, nor yet migratory; it was, in fact, a veritable tramp, without means, and consequently without the power of carrying out its own plans, or its own will, if it had any; it simply waited for an invitation from some local society which was willing to grant to it an asylum for two or three days, and then in due course the meeting was held and another invitation mooted.

The necessary expenses of a meeting of the Association are not large, to be sure, but large or small they had to be met, and as the Association had no funds, it was dependent upon the local society for these expenses. Again, the fact that the Association was the guest of the local profession begat the idea of an entertainment of some kind which, while not burdensome upon the profession of larger cities, was so onerous that the faculty of the smaller places shrank from assuming the obligation, and, as a consequence, the Association has held its meetings in but seven or eight places in the last twenty years.

During the period spoken of it would have been impracticable to locate the Association, because there was no money in the treasury wherewith to pay the necessary expenses, and the burden would have fallen too heavily upon any one local society. In good truth, however, it might almost be said to have a home in Atlanta, for there is a standing invitation from that enterprising city to meet there without further invitation, whenever the Association feels so disposed, or has no other place to meet, and so the Association cannot be said to be without a refuge in case of necessity. Thanks to Atlanta.

### THE ASSOCIATION OF THE PRESENT.

But matters are now changed. By the very satisfactory arrangements made with THE ATLANTA MEDICAL AND SURGICAL JOURNAL, it is probable, nay, almost certain, that the Association will have in the treasury a surplus, small to be sure, but sufficient to meet the necessary expenses attending the meetings of the Association; it need, therefore, be no longer a burden to anyone, whether it is located or not. It need no longer stand knocking at the door, seeking the shelter of a hospitable roof; but, being in a position to pay its own way, it can locate or migrate, as may be determined to be most conducive to its interests.

### THE JOURNAL AS A MEDIUM OF INTERCOMMUNICATION.

It is evident, from what has been said above, that this is a most opportune time to discuss the future policy of the Association as to the manner of selecting its place of meeting, and in the next number of THE JOURNAL I propose to enter upon the subject, unless the medium of discussion suggested should be deemed inappropriate by other members of the committee, in which case, I will, of course, adopt any other method that to them may seem best. If, therefore, I do not hear from any of them within a reasonable time—say two weeks after the publication of this number of THE JOURNAL—I will take the liberty of construing their silence into an acquiescence in the suggestion of THE JOURNAL for the interchange of ideas upon the business submitted to the committee.

Respectfully,

R. J. NUNN, M. D.

*Savannah, Ga., June 15th, 1885.*

